# ﻿Two new species of *Papiliotrema* (Rhynchogastremataceae, Tremellales) from China

**DOI:** 10.3897/mycokeys.126.163715

**Published:** 2025-12-16

**Authors:** Wan-Li Gao, Chun-Yue Chai, Qiu-Hong Niu, Feng-Li Hui

**Affiliations:** 1 School of Life Science, Nanyang Normal University, Nanyang 473061, China Nanyang Normal University Nanyang China; 2 Research Center of Henan Provincial Agricultural Biomass Resource Engineering and Technology, Nanyang Normal University, Nanyang 473061, China Nanyang Normal University Nanyang China

**Keywords:** Basidiomycetes, phylogeny, phylloplane yeasts, taxonomy

## Abstract

*Papiliotrema* species are commonly found in different habitats and many of them are reported as epiphytic yeasts associated with plants. In this study, several yeast strains were isolated from the surface of plants collected in the Chinese provinces of Guizhou and Hainan. Phylogenetic analyses of combined ITS and LSU sequence data placed them in *Papiliotrema.* Two new species, *Papiliotrema millettiae***sp. nov.** (holotype CICC 33641) and *P. mussaendae***sp. nov.** (holotype CICC 33595), are proposed on the basis of phylogenetic analyses and phenotypic characterisation. Detailed descriptions of both species are provided, allowing clear differentiation from other known species in the genus *Papiliotrema*. In addition, *P. fudaokuniae* is considered a synonym of *P. fusca*, based on our phylogenetic analyses. This study contributes to a better understanding of species diversity within the genus *Papiliotrema* and provides a framework for future taxonomic research in the group.

## ﻿Introduction

*Papiliotrema*, a dimorphic yeast genus, was erected by Sampaio et al. (2002) to accommodate the sexual morph *Papiliotrema bandonii* J.P. Samp., Gadanho, M. Weiss & R. Bauer as the type species. Phylogenetically, *P. bandonii* is situated within the *Bulleromyces* clade of the order Tremellales, class Tremellomycetes (Sampaio et al. 2002). This clade comprises teleomorphic genera including *Bulleromyces*, *Papiliotrema* and *Auriculibuller*, as well as several species from the polyphyletic anamorphic genera *Bullera* and *Cryptococcus* (Boekhout 2011). In accordance with the International Code of Nomenclature for algae, fungi and plants and the ‘one fungus, one name’ principle (McNeill et al. 2012), the second species *P. siamensis* Suruss & Limtong was later described, based on two anamorphic strains (Surussawadee et al. 2014). In a major taxonomic revision of basidiomycetous yeasts, Liu et al. (2015) transferred 20 anamorphic species to *Papiliotrema*, including 17 previously classified in *Cryptococcus* and three in *Bullera*, all phylogenetically related to *P. bandonii*. This revision also included the transfer of the sexual species *Auriculibuller fuscus* J.P. Samp, J. Inácio, A. Fonseca & J.W. Fell to *Papiliotrema*. Consequently, the genus now includes more than 20 species, classified in the family Rhynchogastremataceae within the order Tremellales (Liu et al. 2015). The genus has since expanded with the addition of several new species, including *P. odontotermitis* S. Handel, T. Wang, A.M. Yurkov & H. Koenig (Handel et al. 2016), *P. leoncinii* D.M. Pagani, L.R. Brandão, A.R.O. Santos, C.R. Félix, J.P. Ramos, L. Broetto, G. Scorzetti, J.W. Fell, C.A. Rosa, P. Valente & M.F. Landell and *P. miconiae* J.W. Fell, G. Scorzetii & M.F. Landell (Pagani et al. 2016), *P. plantarum* P. Into, A. Pontes, N. Jacques, S. Casarég, S. Limtong & J.P. Sampaio (Into et al. 2018), *P. phichitensis* P. Khunnamwong, J. Surussawadee, N. Srisuk, C. Boonmak & S. Limtong (Khunnamwong et al. 2018), *P. zeae* A.M. Yurkov & C.P. Kurtzman (Yurkov and Kurtzman 2019), *P. horticola* A.V. Kachalkin, A.M. Glushakova & M.A. Tomashevskaya (Crous et al. 2021), *P. tapputiae* Y.P. Tan, Bishop-Hurley & R.G. Shivas (Tan et al. 2023), *P. castaneae* Q.M. Wang and *P. catalpae* Q.M. Wang (Jiang et al. 2024) and *P. fudaokuniae* Y.P. Tan, Bishop-Hurley & Marney (Tan et al. 2024).

To date, the genus *Papiliotrema* comprises 33 species. Amongst them, only three – *P. bandonii*, *P. fusca* and *P. plantarum* – have been reported with known sexual morphs (Sampaio et al. 2002; Sampaio et al. 2004; Into et al. 2018). These sexual states are characterised by clavate basidia with transverse septa, which clearly differentiate them from those of *Tremella* species (Liu et al. 2015; Into et al. 2018). The asexual morphs produce pale to brownish-yellow colonies and reproduce by polar budding (Liu et al. 2015). Physiologically, all members of *Papiliotrema* are non-fermentative, contain ubiquinone Q-10 as the major respiratory quinone and are capable of assimilating a variety of carbon sources, but not myo-inositol or nitrate (Liu et al. 2015).

*Papiliotrema* species are widely distributed in diverse environments. The sexual morph of *P. bandonii* has been observed only on the inflorescences of *Cortaderia selloana* (Sampaio et al. 2002; Saluja and Prasad 2007), while the sexual states of *P. fusca* and *P. plantarum* have been recorded exclusively in culture, similar to the sexual forms of the sister genus *Rhynchogastrema* (Metzler et al. 1989; Sampaio et al. 2004; Into et al. 2018). The yeast morphs of *Papiliotrema* species are predominantly associated with plants, especially leaves (Surussawadee et al. 2014; Sylvester et al. 2015; Yurkov et al. 2015; Pagani et al. 2016; Into et al. 2018; Khunnamwong et al. 2018; Jiang et al. 2024; Tan et al. 2024). Additionally, strains of *Papiliotrema* species have also been isolated from soil (Crestani et al. 2009), glacier ice (de Garcia et al. 2012) and even aquatic environments (Fell et al. 2011; Pagani et al. 2016).

Members of the genus *Papiliotrema* have attracted increasing attention due to their diverse biotechnological potential. Amongst them, *P. laurentii* (Kuff.) X.Z. Liu, F.Y. Bai, M. Groenew. & Boekhout was isolated from a wide range of natural, agricultural and anthropogenic environments (de Almeida et al. 2022). *P. laurentii* is known to produce biosurfactants, various enzymes and high levels of intracellular lipids, which are considered valuable feedstocks for fatty acid-derived industrial products (Vieira et al. 2020; Yalçın et al. 2021). Additionally, it can secrete mycocins with different inhibitory ranges, which has been utilised in the biocontrol of phytopathogenic fungi, thereby contributing to the preservation of fruit quality during both pre- and post-harvest stages (Castoria et al. 1997; Yurkov and Golubev 2013). The species also enhances mycorrhizal colonisation, improves nitrogen uptake and promotes plant growth (Moller et al. 2016). Furthermore, *P. laurentii* has demonstrated the ability to degrade polyester and diesel derivatives and to participate in the bioremediation of heavy metals (Hung et al. 2019). Other *Papiliotrema* species also exhibit functional versatility; for instance, *P. terrestris* (Crestani, Landell, Faganello, Vainstein, Vishniac & P. Valente) X. Z. Liu, F.Y. Bai, M. Groenew. & Boekhout produces exopolysaccharides with potential applications in biocompatible materials and wound healing (Hamidi et al. 2023), while *P. flavescens* (Saito) X.Z. Liu, F.Y. Bai, M. Groenew. & Boekhout has been reported to promote plant growth by modulating root architecture and inducing systemic resistance via volatile organic compounds in *Arabidopsis* (Liu et al. 2024). Furthermore, some species, as *P. laurentii* and *P. flavescens*, have also been isolated from clinical specimens and are considered opportunistic pathogens in humans (Fonseca et al. 2011; Zhang et al. 2022).

During our recent surveys of epiphytic yeasts on leaf surfaces in different regions of China, five yeast strains belonging to the genus *Papiliotrema* were obtained. The objective of this study was to determine the taxonomic identity of these strains and to elucidate their phylogenetic relationships within *Papiliotrema*, based on a polyphasic approach including phenotypic characterisation, molecular phylogenetic analysis and ecological data.

## ﻿Materials and methods

### ﻿Sample collection and yeast isolation

Plant leaf samples were collected from two locations in China: the Guiyang Medicinal Botanical Garden, Guizhou Province (26°34'51"N, 106°42'36"E) and Wuzhi Mountain, Hainan Province (18°17'21"N, 109°40'55"E). Yeast strains were isolated from the phylloplane using the washing and dilution method described by Jiang et al. (2024). Fresh leaves were aseptically cut into small pieces and placed into 10 ml sterile centrifuge tubes containing sterile water supplemented with 0.05% (v/v) Tween 80. The tubes were shaken for approximately 10 minutes and the resulting suspension was serially diluted to 10^−2^. Aliquots of 200 μl from each dilution were spread on to yeast extract–malt extract (YM) agar plates (0.3% yeast extract, 0.3% malt extract, 0.5% peptone, 1% glucose and 2% agar) supplemented with 200 μg/ml chloramphenicol to suppress bacterial growth. The plates were incubated at 25 °C for 5–7 days. Distinct yeast colonies were selected and subcultured on fresh YM agar to obtain pure isolates. Purified strains were preserved in 20% (v/v) glycerol at −80 °C for long-term storage.

### ﻿Phenotypic characteriation

Morphological, physiological, and biochemical characteristics were examined, following the standardised protocols outlined by Kurtzman et al. (2011). Colony morphology was recorded after 7 days of incubation at 20 °C on YM agar. Cellular morphology was observed after 3 days of cultivation in YM broth at 20 °C using a LEICA DM2500 microscope (LEICA, Wetzlar, Germany), equipped with LAS V4.13 software. Ballistoconidium formation was assessed using the inverted-plate method (do Carmo-Sousa and Phaff 1962) on corn meal agar (CMA: 2.5% corn meal infusion and 2% agar) at 17 °C. Discharged spores were collected on glass slides after 3 to 14 days and examined microscopically. The potential for sexual reproduction was evaluated on CMA, potato dextrose agar (PDA: 20% potato infusion, 2% glucose and 2% agar) and V8 agar (10% V8 juice and 2% agar). A loopful of cells from each test strain was streaked alone or mixed on the plates and incubated at 17 °C for up to two months, during which time, observations were made biweekly (Jiang et al. 2024). Glucose fermentation was tested in liquid medium using Durham fermentation tubes. Carbon and nitrogen assimilation tests were conducted in liquid media, with starved inocula used for nitrogen assimilation assessments (Kurtzman et al. 2011). Growth at various temperatures (15, 20, 25, 30, 35 and 37 °C) was assessed on YM agar plates. All novel species descriptions and proposed names were registered in the MycoBank database (http://www.mycobank.org; accessed 26 June 2025).

### ﻿DNA extraction, PCR amplification and sequencing

Genomic DNA was extracted from actively growing yeast cultures on YM agar using the Ezup Column Yeast Genomic DNA Purification Kit (Sangon Biotech Co., Shanghai, China), following the manufacturer’s instructions. Two nuclear loci were targeted for amplification: the internal transcribed spacer (ITS) region and the D1/D2 domain of the large subunit (LSU) rRNA gene. Amplifications were performed using the primer pairs ITS1/ITS4 (White et al. 1990) and NL1/NL4 (Kurtzman and Robnett 1998), respectively. PCR reactions were carried out in a final volume of 25 μl, containing 9.5 μl of double-distilled water, 12.5 μl of 2× Taq PCR Master Mix with blue dye (Sangon Biotech), 1 μl of genomic DNA and 1 μl of each primer. The thermal cycling conditions were: initial denaturation at 98 °C for 2 min; 35 cycles of denaturation at 98 °C for 10 s, annealing at 55 °C for 10 s and extension at 72 °C for 15 s; followed by a final extension at 72 °C for 5 min. PCR products were visualised on 1% agarose gels. Amplicons with single, clear bands were purified using the DNA Gel Extraction Kit (Sangon Biotech) and subjected to bidirectional Sanger sequencing at Sangon Biotech Co., Ltd. (Shanghai, China). Consensus sequences were assembled and edited using BioEdit v.7.1.3.0 (Hall 1999). Sequence identities were confirmed via BLASTn searches against the GenBank database. All newly-obtained sequences were deposited in GenBank (https://www.ncbi.nlm.nih.gov/genbank/).

### ﻿Phylogenetic analyses

For the phylogenetic analyses, sequences generated in this study along with additional related sequences derived from the GenBank database, were used (Table 1). Individual loci were aligned separately using MAFFT v.7.110 (Katoh and Standley 2013) with the G-INS-i algorithm, followed by manual refinement to remove ambiguous regions in BioEdit v.7.1.3.0 (Hall 1999). The resulting ITS and LSU alignments were concatenated into a single dataset using PhyloSuite v.1.2.3 (Zhang et al. 2020). Maximum Likelihood (ML) analysis was conducted using RAxML v.8.2.3 (Stamatakis 2014) under the GTRGAMMA model with 1, 000 rapid bootstrap replicates to assess nodal support. Bayesian Inference (BI) analysis was performed using MrBayes v.3.2.7a (Ronquist et al. 2012). Six Markov Chain Monte Carlo (MCMC) chains were run for 50 million generations, with sampling every 1,000 generations. The optimal substitution model was selected using MrModelTest v.2.3 (Posada and Crandall 1998). The first 25% of sampled trees were discarded as burn-in and the remaining trees were used to estimate Bayesian posterior probabilities (BPPs) for the clades.

**Table 1.** Information of yeast species and strains used in phylogenetic analyses and their GenBank accession numbers. Sequences newly generated in this study are indicated in bold.

Species	Strain no.	Country	GenBank accession no.	
			ITS	LSU D1/D2
*Papiliotrema anemochoreius*	CBS 10258	South Africa	KY104455	KY108727
*Papiliotrema aspenensis*	CBS 13867	USA	NR_158801	NG_060109
*Papiliotrema aurea*	CBS 318	Japan	NR_130650	NG_148937
*Papiliotrema baii*	PYCC 6523	China	LK023827	LK023766
*Papiliotrema bandonii*	CBS 9107	Portugal	NR_121465	NG_042386
*Papiliotrema castaneae*	YN83-2	China	OP470272	OP470176
*Papiliotrema catalpae*	YN109M3	China	OP470271	OP470175
*Papiliotrema flavescens*	CBS 942	Japan	NR_130696	AB035042
*Papiliotrema fonsecae*	CBS 12692	Svalbard	NR_119972	JN193447
*Papiliotrema frias*	CBS 12693	Patagonia	GU997162	LK023834
*Papiliotrema fudaokuniae*	BRIP 76370a	Australia	NR_199259	NG_244360
*Papiliotrema fusca*	PYCC 5690	Portugal	AF444668	AF444762
*Papiliotrema hoabinhensis*	JCM 10835	Vietnam	AB110695	AB193347
*Papiliotrema horticola*	KBP MSU Y-6685	Russia	NR_182874	MW579431
*Papiliotrema japonica*	CBS 2013	Portugal	NR_155613	NG_057690
*Papiliotrema laurentii*	ATCC 18803	Congo	NR_130670	NG_056281
*Papiliotrema leoncinii*	CBS 13918	Brazil	KP203864	KJ608554
*Papiliotrema mangalensis*	CBS 10870	USA	NR_144816	NG_057803
*Papiliotrema miconiae*	CBS 8358	Brazil	AF444387	AF444698
***Papiliotrema millettiae***	**NYNU 243102**	**China**	**PP837692**	**PP837690**
***Papiliotrema millettiae***	**NYNU 24472**	**China**	**PV823290**	**PV823291**
***Papiliotrema mussaendae***	**NYNU 23248**	**China**	**OQ851892**	**OQ851890**
***Papiliotrema mussaendae***	**NYNU 232142**	**China**	**PV823294**	**PV823293**
***Papiliotrema mussaendae***	**NYNU 23229**	**China**	**PV823295**	**PV823292**
*Papiliotrema nemorosa*	CBS 9606	Russia	NR_137534	NG_058365
*Papiliotrema odontotermitis*	CBS 14181	Germany	NR_156605	KU883278
*Papiliotrema perniciosa*	CBS 9605	Russia	NR_137653	NG_060063
*Papiliotrema phichitensis*	DMKU-SP105	Thailand	AB915388	AB826437
*Papiliotrema plantarum*	CBS 15220	Thailand	NR_164566	LC370335
*Papiliotrema pseudoalba*	CBS 7227	Japan	NR_073231	NG_058276
*Papiliotrema rajasthanensis*	CBS 10406	India	NR_155678	NG_058366
*Papiliotrema ruineniae*	PYCC 6170	Indonesia	LK023826	LK023764
*Papiliotrema siamensis*	CBS 13330	Thailand	NR_155608	NG_060062
*Papiliotrema taeanensis*	CBS 9742	Korea	NR_155679	AY422719
*Papiliotrema tapputiae*	BRIP 75038a	Australian	NR_187102	NG_229127
*Papiliotrema terrestris*	CBS 10810	USA	NR_073350	NR_073350
*Papiliotrema wisconsinensis*	CBS 13895	USA	NR_160324	NG_060134
*Papiliotrema zeae*	DSM 104035	Minnesota	NR_168772	MH718306
‘*Cryptococcus*’ sp.	BI311	Brazil	KR063666	KT318492
*Rhynchogastrema aquatica*	CBS 12527	Brazil	NR_120001	NG_042603
*Rhynchogastrema complexa*	CBS 11570	Brazil	NR_111476	NG_042525
*Rhynchogastrema coronatum*	DSM 28188	Germany	LN870267	LN870267
*Rhynchogastrema fermentans*	CBS 12399	Taiwan, China	NR_155732	NG_058388
*Rhynchogastrema glucofermentans*	CBS 10381	Panama	NR_119978	NG_042404
*Rhynchogastrema nanyangensis*	CBS 12474	China	NR_166792	JN564592
*Rhynchogastrema noutii*	CBS 8364	Brazil	NR_111072	NG_042389
*Rhynchogastrema tunnelae*	CBS 8024	Finland	NR_111074	NG_042390
*Naematelia aurantia*	CBS 6965	China	AF444315	AF189842
*Naematelia encephala*	CBS 8207	Germany	AF042402	AF042220
*Kwoniella mangrovensis*	CBS 8507	Bahamas	AF444646	AF444742

Phylogenetic trees were visualised using FigTree v.1.4.3 (Andrew 2016) and final graphical editing was conducted in Adobe Illustrator CS v.5. Branches with bootstrap support (BS) values ≥ 50% and Bayesian posterior probabilities (BPPs) ≥ 0.95 were regarded as statistically significant.

## ﻿Results

### ﻿Molecular phylogeny

In this study, a total of 76 yeast strains were isolated from 25 leaf samples collected. Based on the identification of the ITS and LSU sequences, the majority of these yeast strains were identified as 18 known species, including *Bannoa ogasawarensis*, *Bullera mrakii*, *Bulleribasidium pseudovariabile*, *Cystobasidium pallidum*, *Derxomyces komagatae*, *Dioszegia hungarica*, *Erythrobasidium hasegawianum*, *Filobasidium magnum*, *Hannaella sinensis*, *Hannaella taiwanensis*, *Rosettozyma cystopteridis*, *Sporobolomyces carnicolor*, *Sporidiobolus pararoseus*, *Sporobolomyces roseus*, *Symmetrospora gracilis*, *Symmetrospora oryzicola*, *Tilletiopsis washingtonensis* and *Vishniacozyma carnescens*. The remaining five strains, NYNU 243102, NYNU 24472, NYNU 23248, NYNU 232142 and NYNU 23229, which could not be identified as any known species, were selected for further taxonomic study.

**Figure 1.**
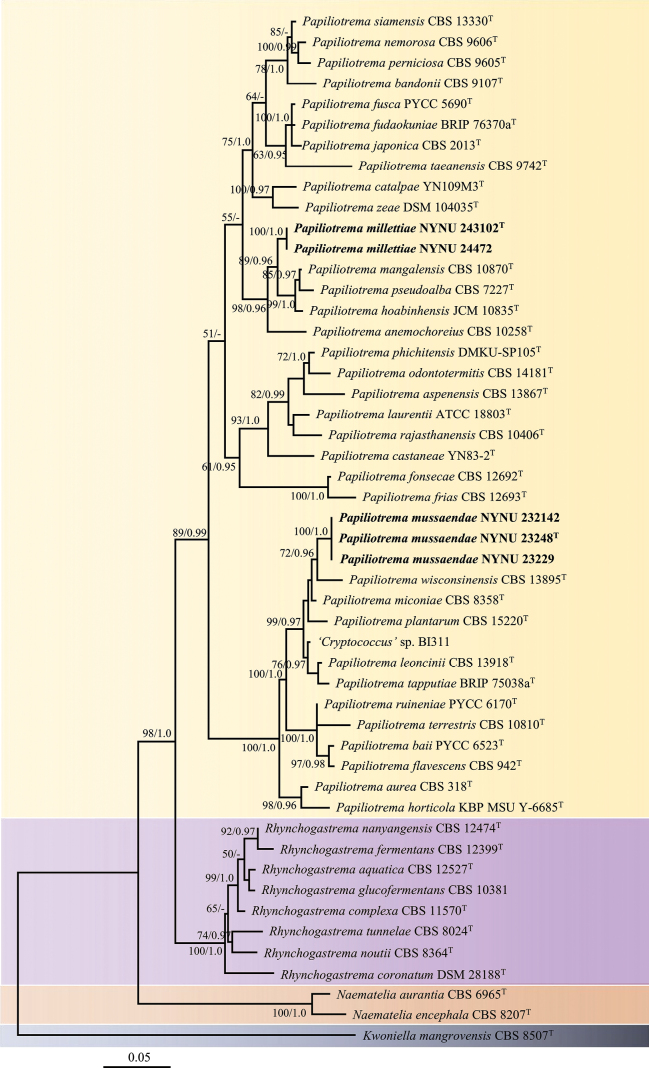
Phylogram of *Papiliotrema*, based on the concatenated ITS-LSU dataset, with *Kwoniella mangrovensis* CBS 8507 as the outgroup. Bootstrap values (BS) ≥ 50% and Bayesian posterior probabilities (BPPs) ≥ 0.90 are indicated at the nodes. Newly-generated sequences are highlighted in bold. “T” denotes sequences from type strains.

To determine the phylogenetic placements of these potential novel strains, phylogenetic analysis was conducted using the concatenated ITS and LSU sequences. The concatenated ITS and LSU dataset included 50 ITS and 50 LSU sequences from 50 strains, representing 46 ingroup taxa plus one outgroup (Table 1). The aligned dataset length was 1,096 nucleotides, comprising 491 from ITS and 605 from LSU region. MrModelTest identified GTR+I+G as the best-fit nucleotide substitution model for BI analysis. BI analysis produced tree topologies nearly identical to those from the ML analysis. Only the ML tree is shown in Fig. 1, with BS (≥ 50%) and BPPs (≥ 0.95) indicated on branches. Phylogenetic analyses revealed that five isolates from China clustered into two genetically distinct lineages, each representing a putative novel species within the genus *Papiliotrema*.

Two strains, NYNU 243102 and NYNU 24472, were isolated from leaves of *Millettia pachycarpa* Benth. and *Musa nana* Lour., respectively, but shared identical D1/D2 and ITS sequences, indicating conspecificity. These strains formed a distinct clade clustering with *P. mangalensis*, *P. pseudoalba*, *P. hoabinhensis* and *P. anemochoreius* in the concatenated ITS and LSU phylogeny (Fig. 1). They differed from these species by 13–17 substitutions (~ 2–2.8%) in the D1/D2 domain and 12–16 mismatches (~ 2.4–3%) in the ITS region, which are higher than the basidiomycetous yeasts thresholds of 0.49% for D1/D2 and 1.39% for ITS, recommended by Vu et al. (2016). These results support that strains NYNU 243102 and NYNU 24472 represent a novel *Papiliotrema* species, for which the name *Papiliotrema millettiae* sp. nov. is proposed.

Three strains, NYNU 23248, NYNU 232142 and NYNU 23229, were isolated from three different leaf samples of *Mussaenda pubescens* Ait.f. and shared identical D1/D2 and ITS sequences, indicating conspecificity. These strains were closely related to *P. wisconsinensis* (Fig. 1) and differed from it by seven substitutions (~ 1.3%) in the D1/D2 domain and 10 mismatches (~ 2.2%) in the ITS region, respectively. Additionally, they also differed from other closely-related species, *P. miconiae* and *P. plantarum*, by 7–8 nucleotide substitutions (~ 1.3–1.4%) in the D1/D2 domain and 10–16 substitutions (~ 2.2–3.6%) in the ITS region. These results support that the three strains represent a novel *Papiliotrema* species, for which the name *Papiliotrema mussaendae* sp. nov. is proposed.

### ﻿Taxonomy

#### ﻿ Papiliotrema millettiae

Taxon classificationFungiTremellalesRhynchogastremaceae

C.Y. Chai & F.L. Hui sp. nov.

BE622B64-F48F-5E44-B031-A997A41DF6D9

859778

Fig. 2

##### Etymology.

The specific epithet *millettiae* refers to *Millettia*, the plant genus from which the type strain was isolated.

##### Typus.

China • Guizhou, Pingtang County, Sifangjing Village, 25°7'N, 107°2'E, on the phylloplane of *Millettia pachycarpa*, Feb 2024, D. Lu, NYNU 243102 (holotype CICC 33641 preserved in a metabolically inactive state, metabolically inactive ex-type culture PYCC 10057), GenBank Accession No.: PP837692 (ITS), PP837690 (LSU).

##### Description.

On YM agar after 7 days at 20 °C, the streak culture is white-cream, butyrous and smooth, with an entire margin. After 3 days in YM broth at 20 °C, cells are ovoid and ellipsoidal, 3.5–6.4 × 4.3–8.9 μm and single, budding is polar. After 1 month at 20 °C, a ring and a sediment are present. In Dalmau plate culture on CMA, pseudohyphae and hyphae are not formed. Sexual structures are not observed on PDA, CMA or V8 agar. Ballistoconidia are not produced. Ballistoconidia are not produced. Glucose fermentation is absent. Glucose, inulin (weak and delayed), sucrose, raffinose, galactose, lactose, trehalose, maltose, melezitose, methyl-α-D-glucoside, cellobiose, salicin, L-rhamnose, D-xylose, L-arabinose, D-arabinose, 5-keto-D-gluconate, D-ribose, ethanol, glycerol, ribitol, galactitol, D-mannitol, D-glucitol, myo-inositol, DL-lactate, succinate, citrate (delayed), D-gluconate, D-glucosamine (weak), N-acetyl-D-glucosamine (weak), 2-keto-D-gluconate, D-glucuronate and glucono-1,5-lactone are assimilated as carbon sources. Melibiose, L-sorbose, methanol and erythritol are not assimilated. Ethylamine and L-lysine and cadaverine (delayed) are assimilated as nitrogen sources. Nitrate and nitrite are not assimilated. Growth is observed at 20 °C and 25 °C, but not at 30 °C. Growth on 50% (w/w) glucose-yeast extract agar is positive. Vitamins are not necessary for growth. Starch-like compounds are produced. Diazonium blue B colour and urease reaction are positive.

**Figure 2.**
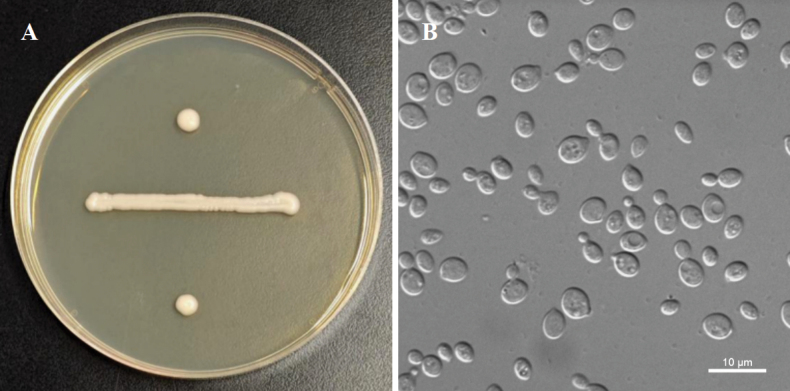
Morphology of *Papiliotrema millettiae* (NYNU 243102). **A.** Colony on YM agar after 7 days at 20 °C; **B.** Budding cells in YM broth after 3 days at 20 °C.

##### Additional strain examined.

China, Hainan, Sanya City, Wuzhi Mountain, 32°45'N, 113°30'E, on the phylloplane of *Musa nana*, Mar 2024, S.L. Lv, NYNU 24472, GenBank Accession No.: PV823290 (ITS), PV823291 (LSU).

##### Note.

*P. millettiae* is phylogenetically closely related to *P. mangalensis*, *P. pseudoalba*, *P. hoabinhensis* and *P. anemochoreius*. Physiologically, *P. millettiae* differs from *P. mangalensis* in its ability to assimilate inulin, methyl-α-D-glucoside and DL-lactate. Compared to *P. pseudoalba*, it cannot assimilate melibiose or grow at 30 °C. It differs from *P. hoabinhensis* in its ability to assimilate inulin and DL-lactate and to grow on 50% glucose medium. Compared to *P. anemochoreius*, it cannot assimilate melibiose or L-sorbose and cannot grow at 30 °C (Table 2).

**Table 2.** Physiological and biochemical characteristics that differ between the new species and closely-related species.

Characteristics	1	2*	3*	4*	5*	6	7*
*Assimilation*							
Inulin	d, w	–	v	–	+	+	n
Melibiose	–	–	+	–	+	+	+
Methyl-α-D-glucoside	+	–	+	w	–	+	n
L-Sorbose	–	s	–	–	+	+	–
Erythritol	–	s, w	–	–	–	+	n
DL-Lactate	+	–	v	–	w	+	n
Nitrate	–	–	–	–	–	+	–
*Growth tests*							
Growth on 50% glucose medium	+	n	–	w	–	+	–
Growth at 30 °C	–	n	+	+	+	–	+

Note. 1, *P. millettiae*; 2, *P. mangalensis*; 3, *P. pseudoalba*; 4, *P. hoabinhensis*; 5, *P. anemochoreius*; 6, *P. mussaendae*; 7, *P. wisconsinensis.* +, positive reaction; –, negative reaction; d, delayed positive; n, not available; s, slow positive; v, variable; w, weakly positive. All data from this study, except* which were obtained from the original description (Boekhout et al. 2011; Fell et al. 2011; Fonseca et al. 2011; Sylvester et al. 2015).

#### ﻿ Papiliotrema mussaendae

Taxon classificationFungiTremellalesRhynchogastremaceae

C.Y. Chai & F.L. Hui sp. nov.

D98E4CD0-223B-5C13-99BC-94311F02048C

859779

Fig. 3

##### Etymology.

The specific epithet *mussaendae* refers to *Mussaenda*, the plant genus from which the type strain was isolated.

##### Typus.

China • Guizhou, Pingtang County, Sifangjing Village, 25°7'N, 107°2'E, on the phylloplane of *Mussaenda pubescens*, Feb 2023, D. Lu, NYNU 23248 (holotype CICC 33595, preserved in a metabolically inactive state, metabolically inactive ex-type culture PYCC 9975), GenBank Accession No.: OQ851892 (ITS), OQ851890 (LSU).

##### Description.

On YM agar after 7 days at 20 °C, the streak culture is white-cream, mucoid, smooth and shiny, with an entire margin. After 3 days in YM broth at 20 °C, cells are ovoid and ellipsoidal, 3.1–4.7 × 3.4–5.7 μm and single, budding is polar. After 1 month at 20 °C, a ring and a sediment are present. In Dalmau plate culture on CMA, pseudohyphae and hyphae are not formed. Sexual structures are not observed on PDA, CMA or V8 agar. Ballistoconidia are not produced. Glucose fermentation is absent. Glucose, inulin, sucrose, raffinose, melibiose, galactose, lactose, trehalose, maltose, melezitose, methyl-α-D-glucoside, cellobiose, salicin, L-sorbose, L-rhamnose, D-xylose, L-arabinose, D-arabinose, 5-keto-D-gluconate, D-ribose, ethanol (weak), glycerol (delayed), erythritol, ribitol, galactitol, D-mannitol, D-glucitol, myo-inositol, DL-lactate, succinate, citrate, D-gluconate, D-glucosamine, N-acetyl-D-glucosamine (delayed), 2-keto-D-gluconate, D-glucuronate and glucono-1,5-lactone are assimilated as carbon sources. Methanol is not assimilated. Nitrate, nitrite, ethylamine, L-lysine and cadaverine are assimilated as nitrogen sources. Growth is observed at 20 °C and 25 °C, but not at 30 °C. Growth on 50% (w/w) glucose-yeast extract agar is positive. Vitamins are not necessary for growth. Starch-like compounds are produced. Diazonium blue B colour and urease reaction are positive.

**Figure 3.**
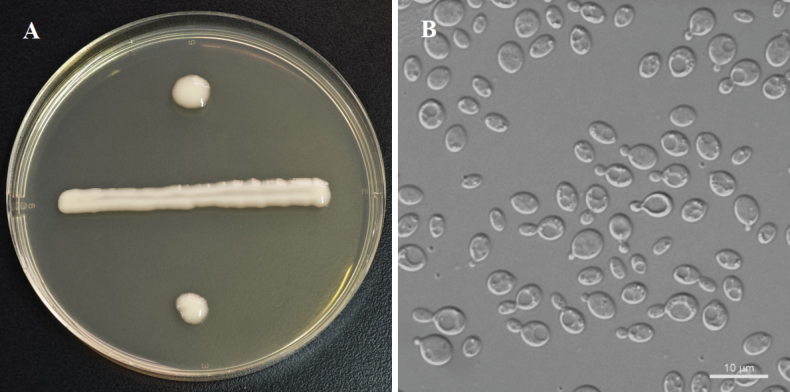
Morphology of *Papiliotrema mussaendae* (NYNU 23248). **A.** Colony on YM agar after 3 days at 20 °C; **B.** Budding cells in YM broth after 3 days at 20 °C.

##### Additional strain examined.

China, Guizhou, Pingtang County, Sifangjing Village, 25°7'N, 107°2'E, on the phylloplane of *Mussaenda pubescens*, Feb 2023, D. Lu, NYNU 232142 and NYNU 23229, GenBank Accession No.: PV823294 and PV823295 (ITS), PV823293 and PV823292 (LSU).

##### Note.

Phylogenetic analyses indicate *P. mussaendae* is most closely related to *P. wisconsinensis*. Both differ in their ability to assimilate L-sorbose and nitrate and its ability to grow at 30 °C from *P. wisconsinensis* (Table 2). Morphologically differs from *P. wisconsinensis* by forming white-cream colonies and smaller cells. Morphologically differs from *P. wisconsinensis* by forming white-cream colonies and smaller cells.

#### ﻿ Papiliotrema fusca

Taxon classificationFungiTremellalesRhynchogastremaceae

J.P. Samp., J. Inácio, Fonseca & Fell ex Haelew.

057199F4-56F4-592D-89DE-CFC2981044B9

836093

= Papiliotrema fudaokuniae Y.P. Tan, Bishop-Hurley & Marney, Index of Australian Fungi 44: 9. 2024.

## ﻿Discussion

In the combined ITS and LSU phylogeny (Fig. 1), *Papiliotrema* is confirmed as a monophyletic genus, consistent with previous studies (Crous et al. 2021; Jiang et al. 20024) with well-supported two clades. The two novel *Papiliotrema* species, described here, are located in each of them. *P. millettiae* forms a distinct lineage within the subclade containing *P. mangalensis*, *P. pseudoalba*, *P. hoabinhensis* and *P. anemochoreius*. *P. mussaendae* clusters with its sister taxon *P. wisconsinensis*.

Several new species have recently been added to *Papiliotrema* (Crous et al. 2021; Tan et al. 2023, 2024; Jiang et al. 2024). Notably, our phylogenetic analyses confirm that the recently proposed *P. fudaokuniae* is closely related to *P. fusca* (Fig. 1). Since *P. fusca* was described earlier, the proposal of *P. fudaokuniae* overlooked the validly described *P. fusca*. These two species share identical sequences in the D1/D2 domain and differ by only four nucleotides in the ITS region, indicating that *P. fudaokuniae* is a synonym of *P. fusca*. Thus, the genus *Papiliotrema* currently includes 34 species, including the two novel species described here.

Although *Papiliotrema* species inhabit diverse environments, most are primarily epiphytic yeasts associated with plant-related substrates, commonly colonising leaves (Khunnamwong et al. 2018). Notably, *P. flavescens* is one of the few species exhibiting genetic heterogeneity amongst the *Papiliotrema* species (Yurkov et al. 2015). It has been reported from distinct locations in Europe, North and South America and Asia and from different sources, primarily from plant-related substrates, such as phylloplane and plant-grazing insects. *P. terrestris*, another widely distributed *Papiliotrema* species, has been found in soil, pigeon droppings and sawdust (Crestani et al. 2009), but a substantial number of isolates are associated with plant material (Yurkov et al. 2015). Additionally, *P. horticola* has been reported as an endophyte from apple hypanthium (Crous et al. 2021), while *P. siamensis* occurs both as an epiphyte and endophyte from plant leaves (Surussawadee et al. 2014). In this study, five strains representing two novel *Papiliotrema* species share a plant-associated ecological niche, consistent with most described species in the genus. *P. millettiae* was isolated from *Millettia pachycarpa* and *Musa nana* in Guizhou and Hainan Provinces, respectively. *P. mussaendae* was repeatedly recovered from *Mussaenda pubescens* in Guizhou Province. The discovery of novel species in previously unexplored regions highlights the need for further surveys to fully characterise the global diversity of *Papiliotrema*. Moreover, investigating their ecological roles, bioactive potential and applications may uncover valuable resources for agriculture, environmental remediation and biotechnology.

## Supplementary Material

XML Treatment for Papiliotrema millettiae

XML Treatment for Papiliotrema mussaendae

XML Treatment for Papiliotrema fusca

## ﻿Additional information

### Use of AI

No use of AI was reported.

### Author ORCIDs

Wan-Li Gao https://orcid.org/0009-0000-1100-7521

Chun-Yue Chai https://orcid.org/0000-0001-7753-6223

Qiu-Hong Niu https://orcid.org/0000-0003-1695-7117

Feng-Li Hui https://orcid.org/0000-0001-7928-3055

### Data availability

All of the data that support the findings of this study are available in the main text.
